# An Introduction to the Influence of Nutritional Factors on the Pathogenesis of Opportunist Fungal Pathogens in Humans

**DOI:** 10.3390/pathogens14040335

**Published:** 2025-03-31

**Authors:** Timothy Jong, Colin M. Stack, Michelle C. Moffitt, Charles Oliver Morton

**Affiliations:** Western Sydney University, School of Science, Campbelltown, NSW 2560, Australiac.stack@westernsydney.edu.au (C.M.S.); m.moffitt@westernsydney.edu.au (M.C.M.)

**Keywords:** virulence, mycosis, aspergillosis, fungal nutrition

## Abstract

Fungi such as *Aspergillus fumigatus*, *Candida albicans*, and *Cryptococcus neoformans* are opportunistic pathogens in humans. They usually infect individuals whose immune system is compromised due to either a primary infection, e.g., HIV/AIDS, or as part of treatment for another condition, e.g., stem cell or solid organ transplant. In hosts with a weakened immune system, these fungi can cause life-threatening infections. Unlike true pathogens, opportunistic pathogens do not have specific mechanisms to overcome a healthy host, requiring a different approach to understand how they cause infection. The ability of fungi to adapt to various environmental conditions, including the human host, is critical for virulence. In humans, micronutrient metals, such as iron, are sequestered to reduce serum concentrations, which helps to inhibit microbial growth. Other human tissues may increase metal concentrations to toxic levels to prevent infection by pathogens. The ability of fungi to acquire or detoxify nutrients, such as iron or copper, from the host is essential for the establishment of infection. In this review, the role of fungal nutrition will be discussed in relation to opportunistic fungal pathogens. It will focus on the acquisition of micronutrients, e.g., iron, copper, and zinc, and how this enables these fungi to circumvent host nutritional immunity.

## 1. Introduction

This short review will act as an introduction to the connections between nutrition and pathogenesis in opportunistic fungal pathogens in humans. An estimated 6.5 million people per year are affected by life-threatening fungal diseases, with the majority caused by opportunist fungal pathogens [1]. The annual incidence of invasive aspergillosis is >1.5 million cases [1]: >1 million for invasive candidiasis and *Candida* septicaemia [1], and >0.5 million for cryptococcosis in individuals with HIV/AIDS [2]. The ubiquity of these fungi in our surrounding environment and an increasing population of susceptible individuals make these fungal infections a serious global health threat.

Fungi are globally distributed and found in most environments, where the majority play an important role as decomposers. Fungi are organoheterotrophs, and cannot synthesise sugars as plants do. They must obtain nutrients from the external environment, principally through the degradation of organic matter [3]. The close proximity between fungi and other organisms can lead to commensalistic, parasitic, or symbiotic interactions. The best example of fungal symbiosis is mycorrhizas, where fungi receive carbohydrates from the plant in exchange for nutrients such as phosphates that the fungi obtain from organic sources in the external environment [4]. Some soil-dwelling fungi can exploit living animals for nutrients, e.g., the nematode-trapping fungus *Arthobotrys oligospora* [5]. This fungus forms rings that constrict around soil nematodes, allowing for the immobilisation and consumption of the nematode. *Pochonia chlamydoporia* can infect the eggs of invertebrates and plant-parasitic nematodes to provide a source of nutrients [6]. Many of these invertebrate pathogens can exploit multiple ecological niches [7]. Fungi can also exploit other fungi for nutrients, including those that infect cultivated mushrooms and cause green mould diseases (*Trichomderma* spp.) or brown spot (*Gliocladium roseum*) [8]. In humans, fungi are dependent on the host as a source of nutrition, being commensalistic or parasitic. The yeast *Candida albicans* can be found in the oral cavity or intestines as a commensal microbe but can also cause potentially life-threating invasive mycosis [9]. Here, we describe the mechanisms that fungi use to obtain nutrients in the environment and how these are important in pathogenesis and overcoming nutritional immunity in the human host.

## 2. Fungal Pathogens

Fungi can be primary pathogens of plants, animals, and even other fungi, causing disease in healthy hosts. Many plant pathogens cause significant losses in agriculture, e.g., the leaf rust of wheat caused by *Puccinia triticina* [10]. There are fungal pathogens of animals, e.g., white-nose syndrome in bats caused by *Pseudogymnoascus destructans* [11] and chytridiomycosis in amphibians caused by *Batrachochytrium dendrobatidis* [12]. These diseases threaten entire animal populations.

A limited number of fungi are primary pathogens in humans, with the most common being those that cause superficial infections, usually referred to as dermatophytes. These typically belong to the genera *Trichophyton*, *Epidermophyton*, or *Microsporum* [13]. These fungi are adapted to the breakdown of keratin, which allows them to exploit the surface of humans as an ecological niche. Although they may cause superficial inflammation, they are restricted to the upper skin layers and do not usually cause serious infection [14]. More serious infections include Paracoccidioidomycosis and Coccidioidomycosis, caused by fungi in the genera *Paracoccidioides* and *Coccidioides*. These fungi are endemic to Central and South America, causing pneumonia in some cases along with cutaneous lesions [15].

### 2.1. Classification of Fungal Infections

In medical practice, fungal infections are classified by the site of infection and the causative agent. In terms of virulence, fungi are classified as either primary pathogens or as opportunistic pathogens. Primary pathogens cause disease in healthy hosts, whereas opportunist pathogens are generally considered to require the host’s immune system to be suppressed either by an existing condition or by medical intervention [16]. However, this is not a strict definition as there are increasing reports of opportunistic mycoses in immunocompetent individuals (discussed further in Section 2.2).

Classification by site of infection recognises superficial, subcutaneous, and invasive infections. Superficial infections are restricted to the surface of the skin or mucosa, with no penetration into the underlying tissues. These are caused by dermatophyte fungi such as *Trichophyton* spp. that infect the upper keratinised layers of skin and *Trichosporon beigleii*, which infects hair [17]. Subcutaneous infections are often caused by the traumatic implantation of spores into the subdermal layers of the skin and underlying tissues. These are often found in individuals who walk barefoot or handle materials that carry infectious spores. These are often present as masses in the subcutaneous tissues that may present with abscesses or as ulcers and can cause tissue destruction. Subcutaneous infections include chromoblastomycosis, caused by *Cladophialophora carrionii*; mycetoma, caused by *Madurella mycetomatis*; and sporotrichosis, caused by *Sporothrix schenckii* [18,19]. Invasive fungal infections often start in the lungs. The spores are usually inhaled and then disseminated from the lungs through the circulatory system, which can lead to the infection of internal organs [20]. These can be caused by primary pathogens such as *Paracoccidioides brasiliensis* or by opportunist organisms such as *Aspergillus fumigatus*. Fungi are morphologically divided into yeasts and moulds; yeasts are unicellular, whereas moulds are multicellular and filamentous. Amongst moulds, there are dermatophytes, which cause superficial infections of skin, nail, and hair, and non-dermatophytes, such as *Aspergillus* spp., that cause superficial, subcutaneous, and invasive infections [16,19,20].

Infections caused by fungi are also named after the causative agent, e.g., *Aspergillus* spp. cause aspergillosis, *Candida* spp. cause candidiasis, and *Mucor* spp. or members of the *Mucorales* cause mucormycosis [20]. Infections caused by less common moulds are termed hyalohyphomycosis (translucent) or phaeohyphomycosis (caused by melanised fungi), referring to their appearance in histopathology samples. Hyalophyomycosis is caused by fungi including *Penicillium* spp. and entomopathogens such as *Paecilomyces* spp., whereas phaeohyphomycosis is caused by fungi such as *Exophiala* spp. and *Cladosporium* spp. [21]. These terms were created from the inability to identify the species using methods where identification can only be made based on fungal morphology. Advances in diagnostic methods such as the use of DNA sequencing and mass spectrometry are increasingly allowing genus- and species-level identification [22].

Some fungi can cause a spectrum of disease in humans. For example, *Aspergillus* spp. can cause infections that can be invasive (invasive aspergillosis, IA) or allergic (allergic bronchopulmonary aspergillosis, ABPA) [23,24]. Aspergilloma refers to the formation of fungal balls in the lung, usually within pre-existing cavities caused by tuberculosis [23]. *Aspergillus* spp. can also cause cutaneous infection, sinusitis, and ocular infections [24].

The names of fungi are increasingly changing due to advances in taxonomy. Mycologists seek to define the taxonomy of fungi based on genetic evidence, which leads to reclassifications of genera and species [25]. This can cause problems in medicine as it leads to changes in the names of common pathogens, e.g., *Candida glabrata* was reclassified to *Nakaseomyces glabrata*, and *Penicillium marneffei* was reclassified to *Talaromyces marneffei* [25]. This has the capacity to cause confusion and has led to debate between mycologists and their colleagues in medicine. Lists of reclassifications of medically important fungi are often published to help scientists and clinicians remain updated with the taxonomy of fungi that cause infection [26,27].

### 2.2. Infections Caused by Opportunist Fungal Pathogens in Immunocompetent Hosts

The most common fungal infections in an immunocompetent host are superficial/cutaneous infections caused by dermatophytes. These are rarely life threatening, but can be a source of morbidity if they are left untreated [28]. There have been cases of dermatophyte infections progressing into the subcutaneous tissues, but this mostly occurred in individuals who were immunocompromised or had a co-morbidity that predisposed them to invasive infection, e.g., diabetes. The overall mortality for individuals with invasive dermatophyte infections was 7.9% [29].

Allergic conditions can occur in individuals who live or work in areas where soils or decomposing plant matter are disturbed; the disturbance releases large numbers of spores that become airborne and can be inhaled by unprotected individuals. Exposure to such conditions and the inhalation of large numbers of spores can lead to farmer’s lung (caused by *A. fumigatus*, *Alternaria* spp.), asthma with fungal sensitisation (caused by *Aspergillus* spp. and *Penicillium* spp.), and allergic rhinosinusitis (caused by *Aspergillus* spp., *Alternaria* spp., and *Cladosporium* spp.) [21].

Although relatively rare, there is an increasing incidence of severe fungal infections in immunocompetent individuals. IA has been reported in gardeners; in these cases, the gardener was exposed to large amounts of conidia at a sufficient inoculum density to overwhelm the pulmonary immune system [30,31].

The Vancouver Island outbreak of *Cryptococcus gattii* led to 218 cases of cryptococcosis in the period from 1999 to 2007, with many of the cases classified as occurring in immunocompetent individuals [32]. Subsequent studies in murine models indicated that *C. gattii* can inhibit the generation of superoxide and neutrophil migration [33]. This contrasts with infection by *C. neoformans*, which does not have the same ability to inhibit the immune function of neutrophils and usually causes infection in immunocompromised individuals [34].

### 2.3. Opportunist Fungal Pathogens

Opportunist pathogens are those that are present in the environment or are a part of the host microbiota, on the skin or mucosal surfaces, that can cause human infection under specific circumstances. An increasing number of fungi that typically act as decomposers in the environment have also been observed to be opportunistic pathogens, capable of causing severe fungal infections [35]. The major requirement for the development of these infections is for the human host to be immunosuppressed or immunocompromised. The most common causes of immunosuppression leading to fungal infections are stem-cell and solid-organ transplants [36]. Immunocompromised individuals typically include those with advanced AIDS or other primary infections such as COVID-19 [37], or tuberculosis [38]. These infections are usually characterised by higher mortality than other fungal infections, but are not as widespread. The major causes of opportunist fungal infections in humans include yeasts (*Cryptococcus neoformans*), moulds (*Aspergillus* spp., fungi of the order *Mucorales*), and tropical-dimorphic fungi (*Candida* spp., *Histoplasma capsulatum*, *Blastomyces dermatidis*, and *Talaromyces marneffei*) [39,40].

## 3. Virulence in Opportunist Pathogens

**Figure 1:**
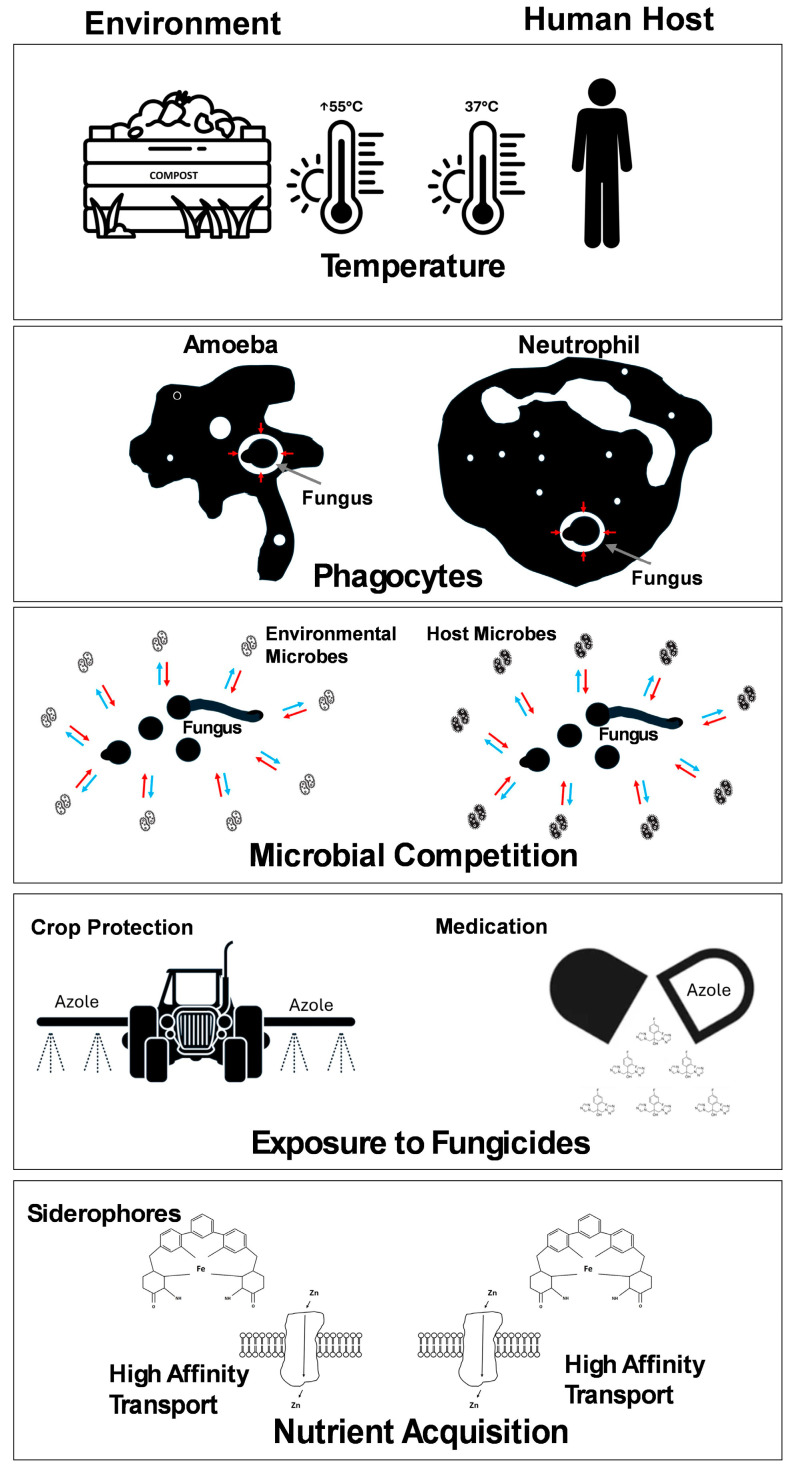
Summary of the challenges faced by opportunist pathogenic fungi in the environment and in the human host. The red and blue arrows in the microbial competition section indicate interactions between the fungal pathogen and environmental/host microbes.

Unlike obligate pathogenic microbes, the majority of opportunistic pathogenic fungi, except those causing tropical mycoses and dermatophytes, do not exhibit the properties of infectious agents. Infections caused by opportunist pathogens are often governed by the immune status of the host. In the immunocompromised, the adaptive immune system is usually disrupted, leaving the innate immune system as the barrier to infection. Characteristics that enable survival in the environment can also be beneficial in adapting to the challenges presented by the host’s innate immune system, which includes body temperature, skin and mucosal surfaces, host microbiota, and macrophages (Figure 1). Using *A. fumigatus* as an example, this organism is often found in organic compost. Compost can reach relatively high temperatures (up to 55 °C); therefore, the fungus has developed thermotolerance, enabling it to survive at temperatures found in the human body (Figure 1) [41]. The fungus has adaptations to withstand predation by soil-dwelling phagocytic protozoa, which has resulted in adaptations that help the fungus to survive encounters with human phagocytic immune cells (Figure 1) [42]. *A. fumigatus* can survive within macrophages and emerge post-phagocytosis, and this is potentially a mechanism that allows the fungus to migrate from the site of initial infection to other host tissues [43]. Furthermore, the soil/compost environment imposes reduced levels of oxygen, creating hypoxic stress, to which the fungus has become adapted. This condition is also found within the human body where the internal organs have reduced oxygen and high CO_2_ levels, and in the lungs, there are regions of localised hypoxia that can be caused by inflammation or disease processes [44]. Adaptation to hypoxia has been linked to virulence in both *A. fumigatus* [44,45] and *C. neoformans* [46]. Fungi show metabolic adaptability with regards to glucose utilisation where fungi can be described as Crabtree-positive or Crabtree-negative. The Crabtree-positive fungi, usually yeasts, downregulate the conversion of pyruvate to acetyl-CoA when there is abundant glucose and switch to fermentation even in the presence of oxygen. The Crabtree-positive fungi upregulate the conversion of pyruvate to acetyl-CoA in order to drive more substrate through the tricarboxylic acid cycle, enabling high yields of ATP [47]. Typically, fungi may switch to ethanolic fermentation during hypoxia to reduce the requirement for oxygen [45]. This is necessary for survival, but it has been observed that hypoxic conditions have very little effect on antifungal sensitivity [48]. The widespread use of azole fungicides in agriculture has provided fungi, within these environments, the opportunity to experience the selective pressure required to develop azole resistance, which could result in fungi that cause human infection (Figure 1) [49]. The emergence of azole resistance in clinical isolates of *A. fumigatus* represents a significant challenge in the treatment of human aspergillosis [50]. *A. fumigatus* can produce secondary metabolites—many of which have antibiotic effects—that are inhibitory to competing microbes or predators, including toxins such as fumagillin and gliotoxin (Figure 1) [51,52]. Finally, the fungus must acquire nutrients from the environment and compete with other organisms for nutritional resources. For example, in low iron (Fe) environments, fungal and bacterial pathogens produce scavenging molecules called siderophores to facilitate the acquisition of Fe from the external environment (Figure 1) [53]. In humans, the resident microbiome found on mucosal surfaces competes with pathogens for resources. This provides an additional form of immunity to infection [54]. However, this protection is not present within the internal organs or bloodstream.

The overall virulence of fungal pathogens has recently been reviewed, giving a broader overview of pathogenesis that is complementary to the current review [55].

## 4. Nutritional Immunity

There is a form of nutritional immunity where essential micronutrients within the human are bound by host factors to reduce their availability to extremely low levels, limiting their availability to pathogens. In the case of Fe, it is mostly bound up in enzymes or to Fe-binding molecules such as ferritin or haemoglobin [56]. This greatly reduces the amount of free Fe available for uptake by pathogens. Fungal siderophores are effective within infected hosts, enabling pathogens to become established (Figure 1). As a result, the ability to acquire nutrients within the host can be considered a virulence factor. The ability to tolerate or overcome starvation within the host is an essential element of pathogenesis. However, nutritional immunity can also take the form of the increased concentration of essential metals, such as copper (Cu), where increased Cu concentrations are associated with the response to infection [57].

## 5. Nutrition and Pathogenesis

The goal of an opportunist pathogen is not to cause disease, but to colonise an environment, extract resources for growth and reproduction, and then disseminate to a new environment. Fungi acquire nutrients through the release of extracellular enzymes that act on their surrounding environment to break down complex substrates and release nutrients [58]. Increased nutrient acquisition can also be achieved through the production of secondary metabolites by the fungus, thereby inhibiting the growth of competing microbes in the environment [52]. The process of extracting nutrients coupled with the release of toxic secondary metabolites can be damaging to host tissues, leading to a disease state involving inflamed or necrotic lesions within tissue and organs [59].

Some of the enzymes released by fungal pathogens are beneficial in overcoming elements of the host’s innate immune system. The enzyme urease, produced by *C. neoformans* and *A. fumigatus*, can modulate the pH of phagolysosomes, preventing their acidification [60,61]. This step is important for the activation of lytic enzymes and their subsequent antimicrobial activity within the immune cell. Once disrupted, the pathogen can survive destruction and exit the immune cell to establish infection.

## 6. Macronutrient Acquisition

The ability of opportunistic fungal pathogens to obtain nutrients is a prerequisite to survival within the host. The major nutrients required, known as macronutrients, include carbon, nitrogen, and phosphorus. However, within the host, nutrient availability varies greatly; the skin is relatively inhospitable to many organisms [62], and even though nutrients are plentiful in the gut, nutrient acquisition can be inhibited by competition with the resident microbiome [63]. Carbohydrate metabolism has been extensively studied in the model yeast *Saccharomyces cerevisiae* and, more recently, in *C. albicans* [64,65,66]. When available, glucose is a simple and preferred source of carbon, with excess glucose stored as glycogen or trehalose. Fortunately, for the human host, many anatomical niches restrict glucose availability and, thus, the ability of opportunistic fungi to cause invasive infection [67]. However, this is not always the case, as evidenced by patients with diabetes in whom yeast infections are more common than in non-diabetic populations due to the reduced control of glucose levels [68,69]. For opportunists to become pathogenic, alternative carbon sources including the utilisation of molecules such as N-acetylglucosamine (GlcNAc) and carboxylic acids (lactate) are required, while proteins can double as a source of nitrogen and carbon [70]. Metabolic adaptability/plasticity is a key driver of infection, providing opportunistic fungi with a fitness advantage and allowing them to cause deep-seated infections, meaning that they should be viewed in the same way as other virulence factors. Furthermore, the central role that glycolysis plays in carbon and energy accumulation is of fundamental importance to survival and is responsible for producing the sugar precursors of the polysaccharides mannan and chitin that are vital for cell wall synthesis. It is therefore unsurprising that targeting carbohydrate metabolism represents an opportunity for therapeutic intervention—specifically, fungal glycogen metabolism. Several disruption studies have demonstrated the importance of glycogen metabolism. The disruption of genes such as glycogen synthase (*gsy1*∆/∆) resulted in long-term survival defects in *C. albicans*, impacting virulence under starvation conditions and in vivo during vaginal colonisation [64]. The same authors found that a global glycogen catabolism mutant (*gph1* ∆/∆ [involved in accumulation], *sga1*∆/∆ [involved in utilisation]) was less virulent during invasive infection, but not lethal, suggesting that alternative carbon sources, e.g., trehalose, were consumed in vivo. A study by Kammer et al. (2020) highlighted the varying survival strategies of the four most common *Candida* species causing bloodstream infections [71]. Their analysis, using human blood, revealed rapid and significant transcriptional changes. These included the downregulation of glycolytic enzymes, the stable upregulation of the alternative glyoxylate cycle, and fermentative energy production. An interesting observation, from a therapeutic perspective, was that each *Candida* species approached the utilisation of alternative carbohydrates differently. The authors suggested that there was no universal fungal virulence strategy among the *Candida* species tested, and that pathogenesis could not be attributed to the regulation of individual genes but was likely more the culmination of several overlapping and redundant responses [71].

While access to these nutrients usually requires the digestion of complex substrates by fungi, this is mediated via the secretion of extracellular protease enzymes [72]. An important family of proteases includes alkaline serine proteases. These play a crucial role in the pathogenesis of invertebrate fungal pathogens, e.g., Pr1 produced by the insect pathogen *Metarhizium anisopliae* [73] and VCP1 from the nematode egg pathogen *P. chlamydosporia* [6]. Proteinase K, produced by the fungus *Parengyodontium album* [74], is a broad-spectrum protease used extensively for the digestion of cell walls, membranes, and tissues in nucleic acid isolation protocols. Its commercial use gives a clue to the role of these proteases in overcoming cellular barriers to infection and the digestion of tissue.

In human infections, *A. fumigatus* produces a serine protease, Alp1, that has a role in digesting the extracellular matrix of the pulmonary tissues [75] and in the development of fungal asthma [76]. An analysis of Alp1 deletion strains indicated that it is not required for virulence because of the upregulation of other proteases to replace its function [77]. It is not the only major serine protease that plays a role in pathogenesis: dipeptidylpeptidases (DPPs) produced by *A. fumigatus* have also been implicated in the binding and digestion of collagen and cytokines, which disrupts immune responses [78]. The DPP proteases are found in spore diffusate, indicating a role in the establishment of growth [79], and induce cytokine production in a murine model [80]. Proteases are important for other opportunistic fungal pathogens. An aspartic protease (Pep1p) was identified in *C. neoformans* that has a role in virulence. It was found that susceptible mice that produced antibodies against Pep1p have improved outcomes [81]. The secreted aspartic proteases of *Candida* spp. are important for several aspects of virulence from biofilm formation to epithelial cell invasion [82,83]. The release of broad-spectrum proteases by fungi in host tissues can also disrupt immune responses by digesting elements of the complement system [84] and eliciting inflammatory responses [85].

Phosphate is the third macronutrient for which environmental fungi have evolved acquisition strategies. In particular, mycorrhiza facilitate phosphate acquisition by plants and this is a central reason for the formation of mycorrhizal associations. The plant can signal phosphate starvation to the fungus, leading to increased phosphate uptake [86]. The main mechanism of phosphate uptake is the PHO system of genes, which was initially characterised in *S. cerevisiae* [87]. PHO-type systems have been identified in *Cryptococcus* [88], *Candida* [89], and *A. fumigatus* [90]. In opportunist pathogens, the PHO-type system becomes necessary for the acquisition of phosphates from host tissues. Phosphate uptake has been associated with virulence in *Candida* [91,92] and *Cryptococcus* [93], with these studies showing the PHO system being highly upregulated in virulence models or through mutational studies. PHO-regulated genes were also identified as upregulated during *A. fumigatus* interaction with dendritic cells [94], as well as evidence of the critical nature of PHO during *C. neoformans* interactions with macrophages [95]. Together, these studies indicate an important relationship between phosphate acquisition and pathogenesis.

## 7. Micronutrient Acquisition

Overcoming the aforementioned nutritional immunity is essential for the establishment of infection. This is achieved by sequestering metal micronutrients or, in some cases, through resistance to toxic levels of Cu. Metals are essential for basic cellular processes, including metabolism, growth, and replication. They play important roles as enzyme cofactors and assist in stress resistance [96]. Studies in several organisms using mutational analysis and infection models have revealed how essential these metals are for virulence.

### 7.1. Iron Acquisition

Fe is a well-studied nutrient associated with fungal pathogenesis. Fe availability is tightly regulated within the host to prevent infection. Fungal pathogens use several mechanisms to overcome this. One response to Fe deficiency is to remodel metabolism to reduce the need for Fe. In *S. cerevisiae*, the most obvious change is a shift from oxidative phosphorylation in the mitochondria, requiring Fe in the cytochromes of the electron transport chain, to fermentation, which is regulated by the mRNA-binding protein Cth2 [97,98]. Cth2 also decreases the expression of other Fe-consuming metabolic pathways such as amino acid biosynthesis and the tricarboxylic acid cycle. These metabolic pathways are also repressed by a Cth2 homolog in *C. albicans* during Fe deprivation [99,100].

In pathogens, three major mechanisms are important for Fe uptake: the production of siderophores to chelate Fe, the heme uptake pathway, and the reductive Fe assimilation system. Pathogenic fungi actively try to scavenge Fe from their growth environment using siderophores. In *A. fumigatus*, it was found that two extracellular siderophores, triacetylfusarinine C and desferriferricrocin, were necessary for virulence; this was achieved through the deletion of the L-ornithine-N*5*-monooxygenase (*SidA*) gene that catalyses the first step in siderophore biosynthesis [101]. In *C. albicans*, a transporter for Fe from siderophores was identified as important for the invasion of epithelial cells, indicating the importance of siderophore-scavenged Fe in pathogenesis [102]. Unlike *A. fumigatus*, *C. albicans* can cause septicaemia [103] and, in this environment, *C. albicans* uses other Fe sources [102]. In the bloodstream, *C. albicans* can lyse erythrocytes and has cell surface receptors for haemoglobin, which is taken up and digested to provide a source of Fe [104,105,106]. *C. neoformans* has been observed to use haemoglobin as a source of Fe with uptake through endocytosis, via the Chc1 protein, located putatively in the trans-Golgi network or endocytic vesicles [107]. The deletion of the *chc1* gene resulted in the inability to incorporate Fe from hemin or haemoglobin. There was some indication that *chc1* and other genes engaged in clathrin-mediated endocytosis have relevance to virulence because the deletion mutants lost the ability to grow at 37 °C and displayed reduced capsule size, and there was a reduction in melanin production; these traits have been corelated with virulence [107,108]. Within the oral cavity, epithelial cells store Fe as ferritin, and hyphal *C. albicans* can use this as a source of Fe during pathogenesis. While the mechanism of extracting Fe from the ferritin protein is not known, it is proposed that the binding of ferritin occurs via the surface protein Als3, with subsequent acidification and uptake of the released Fe via the reductive Fe system [109]. A similar system of host Fe acquisition has been proposed for *C. neoformans*. Macrophages that have phagocytosed *C. neoformans* show increased concentrations of intracellular ferritin, and an ability to extract Fe from ferritin would contribute to the survival of the fungus in the phagolysosome [110,111]. Identified in *C. albicans* but conserved among pathogenic fungi are the ferric reductase FRE1 and metal transporter SMF11 [112]. One major transcriptional regulator responsible for the response to Fe limitation in pathogens such as *A. fumigatus* and *C. neoformans* is HapX [113,114]. HapX leads to the upregulation of siderophore biosynthesis, and its deletion can result in virulence attenuation [113]. In yeast, the Fe response is regulated by Aft1 and Aft2 transcription factors [115].

An interesting case underscoring the importance of Fe in fungal pathogenesis was the increased incidence of Mucormycosis in patients receiving the Fe-chelating drug deferoxamine [116]. The *Rhizopus* spp. that caused the infection, which do not typically grow in human serum, showed enhanced growth when deferoxamine bound with Fe was present. It was subsequently demonstrated that the drug was acting as an Fe carrier for the fungus [117]. Other therapeutic Fe-chelating agents developed and incapable of being used as siderophores by *Mucorales* fungi have been shown to be protective in animal infection models [118].

### 7.2. Zinc Acquisition

Like Fe, limiting zinc (Zn) in the host can be an important defence mechanism against pathogens (nutritional immunity). Zn is required by all organisms as an important part of structural and catalytic proteins, with approximately 10% of the proteome requiring Zn [119]. Zinc-finger domain-containing proteins are the most abundant proteins displaying a range of biological functions, including protein degradation, signal transduction, and transcriptional regulation, with the structure of these proteins being stabilised by the Zn ions [120]. During infection, macrophages display decreased concentrations of Zn as a protective mechanism [121], and fungal virulence factors associated with overcoming Zn nutritional immunity in macrophages likely evolved from interactions with phagocytic amoebas such as *Acanthamoeba castellanii* [122]. Zn also plays an important role in the development of biofilms in the respiratory pathogen *H. capsulatum* [123].

A range of transporters are important in Zn homeostasis [119]. In *C. gattii*, Zap1P, which is highly conserved across the fungal kingdom, regulates the expression of three transporters, Zip1, Zip2, and Zip3 [124], with Zip 1 and 2 being important for Zn transport and virulence in *Cryptococcus* infection models [125]. The deletion of *Zap1* in *C. gattii* led to an increase in reactive oxygen species (ROS) accumulation within the cells, causing increased oxidative stress [125]. The *Zip3* gene is not involved in Zn homeostasis, although it is also important in ROS metabolism and stress response [126]. A second regulatory protein associated with Zn starvation, Zrg1, has been characterised in *C. gattii* and is conserved among *Cryptococcus* spp. [127]. Zrg1 regulates the expression of Zap1P and Zip1 and has also been found to regulate autophagic processes. Autophagy allows *C. gattii* to degrade host Zn-binding proteins, allowing the Zn to be redistributed to essential fungal Zn-binding proteins [127].

The genome of *A. fumigatus* encodes eight transporter genes associated with Zn uptake and regulation. Three of these transporters, ZrfA, ZrfB, and ZrfC, are regulated by the Zn homeostasis transcription factor ZafA [128] and have been examined [129,130]. ZrfA and ZrfB are important in Zn transport under acidic conditions [129,130]. ZrfC assists in the transport of Zn via the 310 amino acid protein Aspf2, an extracellular Zn scavenger, which acts in the same way as a siderophore [129,131,132]. Unlike siderophores, which are typically small molecules, the Aspf2 Zn scavenger (zincophore) is an extracellular protein. The Zn transporter *zrfC* was also expressed in the presence of the Zn/Mn-chelating calprotectin, released by neutrophils, as another mechanism by which Zn acquisition occurs in the face of the host immune response [133]. This distinction is noteworthy, as the bioavailability of Zn atoms in the interstitial spaces of host tissues is estimated to be six to seven magnitudes lower than in host cells [128].

*C. albicans* utilises orthologs of the *A. fumigatus* zincophore and transporter [134]. The Zn scavenger Pra1 is a protein identified in the secretome of *C. albicans*. Pra1 bound to Zn then interacts with the Zn transporter Zrt1 [134]. Similarly, candidalysin produced by *C. albicans* is important for Zn acquisition within the host [135]. Candidalysin is produced during the infection of epithelial cells, causing toxicity to the host and enabling the penetration of host cells where candidalysin sequesters Zn.

#### Zinc in Transcription Factors

Transcription factors can act on several genes, allowing adaptation to environmental signals, and Zn plays an essential role in the functioning of transcription factors [136]. Zn-finger transcription factors are common amongst fungi and control processes from filamentous growth in *C. albicans* by CZF1 [137] to protease expression in *A. fumigatus* through PrtT [138]. In opportunistic pathogenic fungi, there are >300 Zn-finger transcription factors in *A. fumigatus*, >100 in *C. albicans*, and >100 in *C. gattii* and *C. neoformans* [139]. In *A. fumigatus*, PacC is linked to the response to environmental pH and is also linked to host tissue invasion [140]. The PacC protein is a Zn-finger transcription factor that binds Zn atoms, allowing further interaction with DNA to regulate gene expression [141]. In *C. neoformans*, zinc-finger-containing transcription factors STE12α and STE12a are associated with the mating strains MATα and MATa, respectively. These STE12 transcription factors are important for virulence in *C. neoformans*, regulating capsule, phospholipase, and superoxide dismutase gene expression, all important factors in virulence [142,143]. Fe homeostasis is also regulated by the Zn-finger transcription factor Cir1 in *C. neoformans* [144]. These activities further reinforce the importance of Zn acquisition to pathogenic fungi.

### 7.3. Copper Acquisition

**Figure 2:**
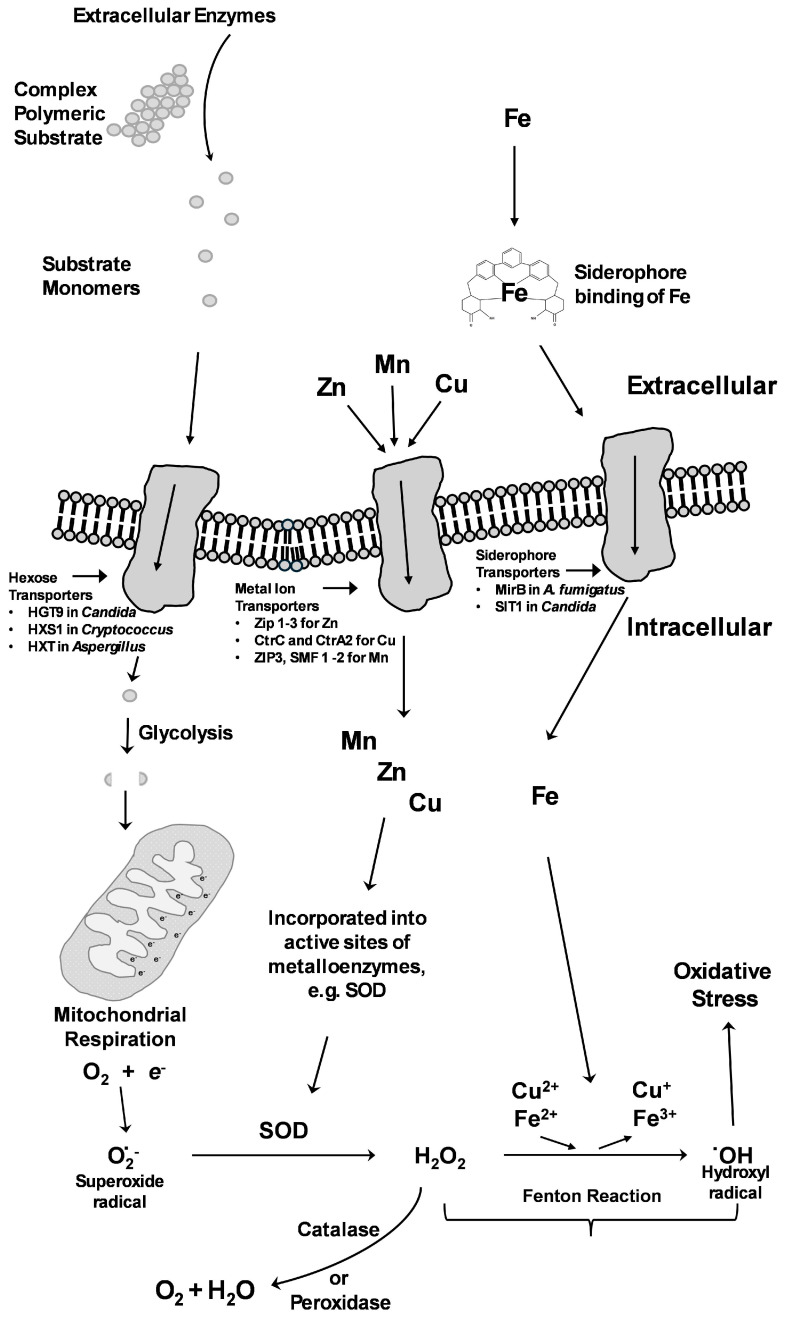
Relationship between metal micronutrients and oxidative stress. Metal micronutrients and macronutrients, such as glucose, must be acquired from the environment and then imported into the cell. Respiration leads to the production of superoxide radicals, and these are detoxified by the enzyme superoxide dismutase (SOD), which requires Mn, Cu, or Zn as co-factors. Production of hydrogen peroxide by this reaction can react with excess Fe or Cu, leading to the Fenton reaction, which produces hydroxyl radicals leading to oxidative stress.

As with other metal micronutrients, Cu is an important enzyme cofactor facilitating mitochondrial electron transfer reactions, as well as the biosynthesis of melanin [145]. In *A. fumigatus*, Cu uptake is mediated by the transcription factor MAC1, which regulates four Cu transporters [146,147]. The transporters CtrC and CtrA2 are crucial, working synergistically under the conditions of Cu scarcity, and the deletion of *mac1* leads to reduced pathogenicity in murine infection [146]. There is also overlap between the regulation of Zn and Cu homeostasis, with the Zn transcriptional regulator ZafA being able to affect Cu metabolism through the upregulation of *CtrC* and *CtrA2* under the conditions of Zn starvation [148]. In *C. albicans*, in addition to the transporter CtrI, the ferric reductase Fre7 aids in uptake by reducing Cu, while the manganese (Mn) transporter SMF12 may also play a role in Cu transport [149]. MAC1 is also essential for Cu homeostasis and virulence in *C. albicans*, with deletion mutants displaying reduced infection in murine models and deficient mitochondrial respiration [150]. The *C. albicans macI* deletion mutant was also hypersensitive to ergosterol inhibitor fungicides, and was rescued by the addition of Cu to the media [149]. These deletion mutants have demonstrated that MAC1 is integral for host cell adhesion, although Cu supplementation to the media did not reverse the effect, indicating that MAC1 regulates other genes associated with adhesion not directly associated with Cu homeostasis [149]. MAC1 has a similar role in *H. capsulatum*, regulating the Cu transporter gene *CTR3* and other genes important for the infection of phagocytes, with the deletion of *mac1* decreasing intracellular macrophage proliferation [151]. Once transported across the membrane to the cytosol of the fungal pathogen, free Cu must be bound to prevent uncontrolled Fenton reactions (Figure 2) and non-specific binding to proteins. In the yeasts *S. cerevisiae*, *N. glabrata*, and *C. neoformans*, cysteine-rich metallothioneins are important in binding free Cu. Metallochaperones and intracellular carrier proteins are also important in transporting Cu to cuproenzymes and mitochondrial proteins or to mediate Cu storage [152].

#### Adaptation to High Copper Concentrations

Overcoming Cu limitation is important for fungal infection; however, high concentrations of Cu are also an important antimicrobial defence used by phagocytic cells such as macrophages [153,154]. Cu has been employed as an antimicrobial surface cleaning agent in healthcare settings where it is used on door handles to reduce fomite transmission [155]. Host Cu concentrations within serum and phagocytic cells have been demonstrated to rise during infection [57,154]. Therefore, understanding the response of fungal pathogens to increased Cu levels is of importance.

In addition to its role in Cu limitation, MAC1 is also important for survival under high Cu concentrations, where it regulates the expression of Cu efflux pumps as well as playing a role in the oxidative stress response (including the expression of catalase and superoxide dismutase (SOD)) in *H. capsulatum* [151]. Both adaptations are necessary for its survival within the host and phagolysosome of phagocytic cells. In *A. fumigatus*, MAC1 is important in regulating the efflux of Cu [146], while another transcription factor, AceA, is also capable of activating Cu efflux and resistance to Cu [156]. *C. neoformans* initiates infection in the lung, where Cu concentrations are high, before moving to the brain where Cu is limited, highlighting the need to understand the response to both high and low Cu concentrations. Within the host, *C. neoformans* possesses Cu-detoxification (*CMT*) genes that encode Cu-binding metallothioneins that are induced in the presence of increased Cu concentrations [157,158]. The deletion of *CMT* genes in *C. neoformans* leads to reduced virulence in a murine infection model [158]. This highlights the importance of understanding Cu homeostasis within the host and fungal pathogen during different stages of infection.

### 7.4. Manganese Acquisition

While a significant number of studies have investigated Fe, Cu, and Zn in pathogenesis and nutritional immunity, less is known about Mn. Like Fe, Mn is an important enzyme cofactor. Mn is also important for SOD activity, a vital response enabling pathogenic fungi to counter the oxidative stress produced as a defence mechanism by the host immune cells as well as during aerobic growth because of the Fenton reaction (Figure 2) [159]. In a murine model of infection, it was found that the deletion of Mn SOD led to a complete loss of virulence in *C. neoformans* [160]. Several catalytic enzymes also require Mn as an essential cofactor, with one of the most important being glycosyltransferases, specifically mannosyltransferases, which are important in processing proteins in the Golgi [161]. In *C. albicans*, a null mutant of Pmr1p, which transports Cu and Mn to the Golgi for transfer to glycosyltransferases, highlighted the importance of Mn-containing glycosyltransferases in maintaining the cell wall and virulence in *C. albicans*. In *C. gattii*, the Mn transporter ZIP3 is proposed to be localised to the membrane of the Golgi [126]. The deletion of *ZIP3* led to imbalanced ROS metabolism, the secretion of the virulence factor GXM, and impaired virulence in a *Galleria mellonella* infection model, which may be the result of reduced cell wall melanisation.

As with other metal nutrients, Mn transporters are key for the survival of fungal pathogens under Mn starvation induced by the host. Interestingly, a study by Wildeman et al. (2023), which employed a mouse model of disseminated candidiasis, with the kidney as the primary target tissue, demonstrated the dramatic loss of total kidney Mn in response to infection, further highlighting the importance of the host’s restriction of this key transition metal [162]. Key to countering this during the invasion of host cells, *C. albicans* employs two natural resistance-associated macrophage protein (NRAMP) transporters, *SMF12* and *SMF13*. The disruption of either gene, individually or in combination (smf12∆/∆ and smf1∆/∆ mutations), results in a dramatic (10–80-fold) reduction in cellular Mn. Furthermore, deletion mutants had impaired mitochondrial Mn-Sod2 and cytosolic Mn-Sod3 activities, defective hyphal formation, and attenuated virulence in a mouse model of disseminated candidiasis [162,163]. As mentioned previously, the Mn transporter SMF12 is also important in Cu starvation [149]. RNA-seq revealed that Mn starvation in *C. albicans* induced metabolic genes and Fe transporters, while the genes for Mn-containing mannosyltransferases and cell wall integrity proteins were downregulated [163]. In these Mn-starved cells, intracellular Fe levels were also reduced, a common phenomenon in other organisms. In addition, the virulence factors required for *C. albicans* to initiate hyphal growth and the production of candidalysin were induced [163]. Interestingly, Mn-replete conditions also moderately increased sensitivity to antifungals. The chelation of Mn by neutrophils, the predominant cell type in corneal ulcers, was mediated by the protein calprotectin, which caused the inhibition of *A. fumigatus* hyphal growth [164]. There is overlap in the uptake of Fe and Mn, with the transcription factor SreA playing an important role in the regulation of Fe uptake in *A. fumigatus* [165], as well as being involved in Mn uptake and tolerance [166].

## 8. Conclusions

The ability to acquire nutrients from the host is essential for the establishment of fungal infections. The limitation of any single macronutrient or metal micronutrient (Fe, Zn, Mn, and Cu) can impact viability and prevent infection, while some human tissues can use metal toxicity as an alternative resistance mechanism. Although pathogens can usually overcome nutritional immunity, the development of agents that can disrupt specific mechanisms of nutrient acquisition or homeostasis within the pathogen could help to prevent fungal infection. Given the limited classes of antifungal drugs available [167], metal ion homeostasis in pathogenic fungi could become a novel target for future antifungal drug development [168,169]. The importance of nutrition in fungal pathogenesis also highlights the necessity of understanding the basic biology of opportunist fungal pathogens in their natural habitats to explain their response to the infected host. Further study of fungal biology, including reproduction and metabolism, has the potential to reveal new avenues for the development of therapeutics or the removal of these pathogens from healthcare settings.

## Data Availability

No new data was generated for this narrative review.
